# Roles of sex and SP-A genetic variants in modulating multiorgan injuries post viral infection

**DOI:** 10.3389/fimmu.2026.1866913

**Published:** 2026-08-21

**Authors:** Zachary M. Williams, Ikechukwu B. Jacob, Akinkunmi O. Lawal, Hongkun Quan, Julia Comprix, Paul T. Massa, Saravanan Thangamani, Guirong Wang

**Affiliations:** 1 Department of Surgery, SUNY Upstate Medical University, Syracuse, NY, United States; 2 Department of Microbiology & Immunology, SUNY Upstate Medical University, Syracuse, NY, United States; 3 Department of Neurology, SUNY Upstate Medical University, Syracuse, NY, United States

**Keywords:** humanized transgenic mice, innate immunity, multiple organ injury, SARS-CoV-2 infection, sex difference, surfactant protein A (SP-A)

## Abstract

**Background:**

Severe acute respiratory syndrome coronavirus 2 (SARS-CoV-2) infection can lead to acute lung injury (ALI)/acute respiratory distress syndrome (ARDS), and multi-organ dysfunction (MOD). Human surfactant protein A (SP-A), a key component of innate immunity, exhibits genetic polymorphisms that may influence host responses to viral infection. Human patient studies have shown that biological sex has been associated with differences in COVID-19 severity. However, the combined effects of SP-A variants and sex on SARS-CoV-2-induced organ injury remain poorly understood.

**Methods:**

We utilized double-humanized transgenic mice expressing human ACE2 and individual human SP-A variants (6A^2^, 6A^4^, 1A^0^, 1A^3^), alongside SP-A knockout (KO) and wild-type (K18) controls. Male and female mice were intranasally infected with SARS-CoV-2 (Delta variant) and assessed on day 6 post-infection. Histopathological analyses were performed to evaluate lung, kidney, and intestinal injury. In parallel, *in vitro* assays assessed SP-A variant binding to viral spike (S) protein and receptor-binding domain (RBD), as well as effects on viral infectivity in lung epithelial cells.

**Results:**

SP-A variants significantly attenuated SARS-CoV-2-induced organ injury compared to KO mice, with SP-A variant and organ-specific effects. In the lung, injury severity followed the order 6A^2^ > 1A^0^ > K18 > 1A^3^ > 6A^4^, with female mice, particularly those expressing 1A^3^ and 6A^4^, exhibiting markedly reduced ALI compared to males. In the kidney, 1A^0^ and 6A^4^ variants showed the greatest protection, again with stronger effects in females. In contrast, intestinal injury was attenuated by SP-A variants independent of sex. Across tissues, SP-A deficiency was associated with increased viral burden and inflammation. Mechanistically, SP-A variants differentially bound SARS-CoV-2 S protein and RBD, with the 1A^0^ variant demonstrating the stronger binding and greater inhibition of viral infectivity compared to 6A^2^ variant *in vitro*.

**Conclusions:**

Human SP-A genetic variants and biological sex interact to modulate SARS-CoV-2-induced multi-organ injury in a tissue-specific manner. These findings highlight the importance of host genetic variation in innate immunity and suggest that SP-A variants, together with sex-specific considerations, may inform risk stratification and therapeutic strategies for COVID-19 and related viral diseases.

## Introduction

There have been nearly 800 billion people infected and over 7 million deaths due to SARS-CoV-2, barely six years since it was first reported (1, 2). Severe acute respiratory syndrome coronavirus 2 (SARS-CoV-2) is an enveloped, single-stranded RNA virus that encodes four structural proteins: the spike protein (S protein), envelope (E), membrane (M), and nucleocapsid protein (N) (3). Viral infectivity is mediated by the trimeric S protein, which is the primary attachment factor to the human angiotensin-converting enzyme 2 receptor (hACE2) expressed on alveolar type 2 cells (AT2) of the lungs, intestines, kidneys, heart, brains, and in several other organs (4). The S protein contains a single polypeptide chain with two subunits (S1 and S2) per monomer. Within the S1 subunit is a receptor-binding domain (RBD) which primarily attaches to hACE2 while the S2 domain mediates the fusion of the viral and host cell membrane (5). Similarly to other enveloped viruses like HIV-1 and influenza A virus (IAV), the SARS-CoV-2 S protein is heavily glycosylated, a modification that has been found to conceal a major portion of its surface from neutralizing antibody recognition (5, 6).

The hallmark of severe COVID-19 is a high viral burden in the lungs and a profound influx of pro-inflammatory cytokines from local and systemic inflammation resulting in acute lung injury (ALI) and acute respiratory distress syndrome (ARDS) (7, 8). Most infected individuals experience mild to moderate febrile illness, but severe illness is marked by ALI/ARDS, respiratory failure, hypoxemia, and death (2, 7, 9). In some infected individuals, dysregulation of host immune responses has been shown to result in enhanced local and systemic levels of proinflammatory cytokines causing long-term multiple organ diseases (MOD) affecting the gastrointestinal, cardiac, and nervous systems alongside virus-induced lung damage (8, 10, 11). While Genome-Wide Association Studies (GWAS) have demonstrated differential disease outcomes in the general population (with some implicating genetic variations in host innate immunity), the reasons for the variability in disease phenotypes are still unresolved (12–17).

Human surfactant protein A (SP-A) is a C-type lectin expressed by epithelial cells of the lungs, kidneys, intestines, and brain where it functions as a first line of defense against invading pathogens (18, 19). As pattern recognition molecules (PRM), collectins such as SP-A, SP-D, and mannose-binding lectins bind to a variety of microbial surface glycoproteins, inhibiting their invasion of susceptible cells to alleviate disease severity (18, 20–22). SP-A mediates bacterial and viral aggregation, opsonization, and lysis while modulating inflammation by binding to various types of activating and inhibitory receptors on immune cells (18, 23). We and other groups have demonstrated the antiviral and immunomodulatory activities of SP-A in the context of SARS-CoV-2, Respiratory Syncytial Virus (RSV), Influenza A Virus (IAV), and Human Immunodeficiency Virus-1 (HIV-1) by binding to viral surface glycoproteins and inhibiting infectivity in host cells (24–27).

However, the SP-A gene is highly polymorphic, with two functional genes: *SFTPA1* (SP-A1) and *SFTPA2* (SP-A2) as well as several variants (alleles) for each of these genes that have been identified in the general population: SP-A1 variants: 6A^2^ and 6A^4^ and SP-A2 variants: 1A^0^ and 1A^3^ (28). Although these variants differ in only in few amino acids, SP-A2 variants have demonstrated higher antimicrobial and immunostimulatory capacity than SP-A1 variants (29–32). SP-A genetic variants have been associated with various types of acute and persistent diseases in the human population (33). Specifically, a study found that SP-A2 (1A^0^) is associated with an increased risk of acute respiratory failure and ARDS following IAV infection while variations in SP-A genes were shown to be either protective or risk factors for severe RSV-induced lung distress (34, 35). Thus, the unique SP-A polymorphism an individual carries can be used as a marker to identify or classify those at risk for certain diseases (36). Recently, our group observed that human SP-A variants modulate SARS-CoV-2 infection and protect against lung injury in our *in vivo* mouse model (37, 38). Therefore, a better understanding of the contributions of human SP-A genetic variants in the context of SARS-CoV-2 infection is essential to develop novel variant-specific surfactant-based therapies for COVID-19.

Meta-analyses have shown a higher prevalence of infection as well as proportion of severe/critical illness and death among human males infected with SARS-Co-V-2 vs their female counterparts (39, 40). Proposed explanations attribute both behavioral and sex-based physiologic immunity differences (39). Females have been shown to have distinct protective differences in innate and adaptive immune responses in both human and animal models due to genetic (specifically immune-modulating genes on the X chromosome) and hormonal factors (protective estrogen/progesterone vs risk-inducing androgens) (41). In both humans and mouse models, females have been shown to have a lower incidence of ARDS/ALI vs their male counterparts (42, 43). Aside from lung infection and injury, male human and animal models have also shown increased risk and severity of gastrointestinal and hematologic infections and less for both urinary tract and sexually transmitted infections (44, 45). We also know that androgens upregulate ACE2 and TMPRSS2, (facilitators of SARS-CoV-2 cellular invasion) in lung epithelial cells on top of testosterone’s role in the suppression of the immune response (46–48), while estrogen downregulates ACE2 in lung and kidney cells along with its anti-inflammatory and immune modulatory effects (49–54). However, there remains a significant gap in research into the roles of sex along with surfactant proteins in modulating immune response to infection.

In this study, we employed double-humanized transgenic mouse models expressing hACE2 and individual human SP-A variants to investigate the combined effects of SP-A genetic variation and biological sex on SARS-CoV-2-induced multi-organ injury. Using both *in vivo* and *in vitro* approaches, we demonstrate that SP-A variants differentially modulate viral infectivity, inflammation, and tissue injury in a variant-, organ-, and sex-dependent manner. These findings provide new insights into the role of innate immune genetic diversity in COVID-19 pathogenesis and identify SP-A variants as potential determinants of disease susceptibility and severity.

## Materials and methods

### Animal models

The mice used for this study were bred and maintained in the animal core facility at SUNY Upstate Medical University. Mice were housed in temperature-controlled room at 22 °C under specific pathogen-free conditions. 10–14 weeks old male and female mice were used in this study. The animal experiments were approved by the Institutional Animal Care and Use Committee of the SUNY Upstate Medical University and were performed according to the National Institutes of Health and ARRIVE guidelines on the use of laboratory animals. The original hACE2/mSP-A (K18) mice were purchased from Jackson Laboratories (Bar Harbor, ME). K18 mice are highly susceptible to SARS-CoV-2 infection with similar pathological outcomes as seen in mild to moderate COVID-19 patients (37). However, K18 mice have a mouse SP-A (mSP-A) background. To study the role of human SP-A (SP-A) variants in the context of SARS-CoV-2 infection, we generated mice that carry both hACE2 and individual single gene variants of either SP-A1 (6A^2^ and 6A^4^) or SP-A2 (1A^0^ and 1A^3^) without mouse SP-A background by breeding with previously characterized hTG SP-A mice (37, 38, 55). Briefly, we first crossed K18 mice that are hemizygous for the hACE2 transgene with our human SP-A knockout (KO) mice to remove mSP-A. Genotyping was performed after each filial generation. Subsequently, we crossed the hACE2/SP-A KO mice with individual variants of either SP-A1 (6A^2^ or 6A^4^) or SP-A2 (1A^0^ or 1A^3^) genes to generate a double-hTG mouse model with hACE2 and SP-A transgenes (38). We examined the presence of hACE2 and SP-A genes in the mice by PCR genotyping analysis using DNAs isolated from mouse tail biopsy and analyzed SP-A expression by Western blotting. The double-hTG mice (hACE2/6A^2^ (6A^2^), hACE2/6A^4^ (6A^4^), hACE2/1A^0^ (1A^0^) & hACE2/1A^3^ (1A^3^) alongside hACE2/SP-A KO (KO) and hACE2/mSP-A (K18) mice were used for subsequent challenge studies with SARS-CoV-2 delta variant (38).

### Mouse infection and sample processing

Mock-challenged and SARS-CoV-2-infected mice (6 groups: 6A^2^, 6A^4^, 1A^0^, 1A^3^, K18 and KO) were anesthetized with isoflurane and infected intranasally (i.n.) with 30 µL (15 µl/nose) of virus suspension containing 1× 10^3^ PFU of SARS-CoV-2 (delta variant) in 1X MEM. Control (Sham) mice were inoculated with 1X MEM. After viral infection, mice were observed daily for morbidity (body weight) and mortality (survival). Mice showing >25% decrease in their initial body weight were defined as reaching the experimental endpoint and euthanized (38). Mice were sacrificed by anesthesia and exsanguination on day 6 post-infection to obtain lung, kidney, intestine samples for viral load analysis by immunohistochemistry (IHC), and plaque assay. Some mice (whole-body) were fixed in 10% neutral buffered formalin (NBF) for further histological and IHC analyses. All experiments were performed with an approved IACUC protocol (IACUC #507) by the IACUC committee of SUNY Upstate Medical University, Syracuse NY. For histological analysis, group size for each SP-A variant and each sex was balanced wherever possible based on the availability of each mouse subtype. Groups were created using samples spanning 5 identical rounds of infection and processing that occurred over the course of 3 years.

### Tissue processing and isolation

Mice were anesthetized with isoflurane and exsanguinated. Then we perfused each mouse with 3 mL NBF. After which the lungs were inflation-fixed using tracheal instillation of 1 mL NBF. Mouse whole bodies were dissected along the abdomen and fixed in NBF before histological and immunohistochemical analysis. Lungs, kidneys, intestines, and brains from a subset of mice were harvested and frozen immediately at -80 °C for further processing.

### Histopathological analysis

Fixed tissues were embedded in paraffins as described previously (37, 56). About 5 µm sections of each tissue sample were cut and stained with Hematoxylin and Eosin (H&E) for light microscopic examination. Histopathological studies were carried out by one pathologist. Experimental groups were created, including both male and female divisions of the four double-hTG mice (6A^2^, 6A^4^, 1A^0^, 1A^3^) alongside SP-A KO and mouse SP-A (K18). These groups were compared first negating sex. Each variant was subsequently broken down by sex to compare each SP-A variant as well as to compare male vs female within each SP-A variant grouping. Specific n for each group can be found in the following methods subsections describing organ injury analysis. Groups were balanced based on the total available number of each mouse subtype by sex and SP-A variant.

### Acute lung injury analysis

Lung injury was assessed using the American Thoracic Society (ATS) scoring system by counting neutrophils in different areas: alveolar space (A, none = 0, 1-5 = 1, >5 = 2) and interstitial space (B, none = 0, 1-5 = 1, >5 = 2), hyaline membranes (C, none = 0, 1 = 1, >1 = 2), proteinaceous debris in airspaces (D, none = 0, 1 = 1, >1 = 2), and alveolar septal thickening (E, none = <2x, 2x-4x = 1, >4x = 2) (57). ALI scores for each field were calculated with the following weighted formula: [(20 x A) + (14 x B) + (7 x C) + (7 x D) + (2 x E)]/(number of fields) x 100. Field scores were averaged to determine the total score for each lung sample (57). N for each group is as follows: SP-A KO = 6 male, 7 female; mSP-A = 6 male, 5 female; 1A^0^ = 3 male, 6 female; 1A^3^ = 7 male, 4 female; 6A^2^ = 5 male, 5 female; 6A^4^ = 6 male, 8 female.

### Acute kidney injury analysis

Kidney injury was assessed by counting the number of tubular cells with vacuolar degeneration, cell necrosis, loss of brush border with dilation of the tubular lumen, and cast formation. Scores for each field were assigned using the following breakdown: 0= none, 1 = 10%, 2 = 11-25%, 3 = 26-45%, 4 = 46-75% and 5= >76% (58). Field scores were averaged to determine the total score for the individual kidney sample. N for each group is as follows: SP-A KO = 4 male, 4 female; mSP-A = 3 male, 3 female; 1A^0^ = 3 male, 3 female; 1A^3^ = 5 male, 3 female; 6A^2^ = 3 male, 3 female; 6A^4^ = 3 male, 3 female.

### Acute intestinal injury analysis

Small intestinal injury was assessed using Chiu’s scoring system (59). Pathological grading of individual fields were performed by evaluating for normal mucosal villi (grade 0), development of subepithelial space with capillary congestion (grade I), extension of the subepithelial space with moderate lifting of the epithelial layer (grade II), massive epithelial lifting down the sides of villi (grade III), denuded villi with lamina propria and dilated capillaries exposed and increased cellularity of the lamina propria (grade IV), and digestion and disintegration of the lamina propria with hemorrhage and ulceration (grade V). Field grades were averaged to determine the total score for the individual intestine sample. N for each group is as follows: SP-A KO = 3 male, 4 female; mSP-A = 3 male, 2 female; 1A^0^ = 3 male, 5 female; 1A^3^ = 4 male, 3 female; 6A^2^ = 3 male, 2 female; 6A^4^ = 4 male, 2 female.

### Human SP-A protein preparation

Native human SP-A (hSP-A) was isolated and purified from bronchoalveolar lavage fluid (BALF) of alveolar proteinosis patients with an approval IRB protocol (IRBNET # 2020-E) as described previously (32). The purity of the SP-A preparation was confirmed by SDS-PAGE followed by silver staining and then filtered through a 0.2-micron filter to remove potential contaminants.

### Purification of human SP-A genetic variants expressed in mammalian cells

Human SP-A genetic variants derived from SP-A1 (6A^2^ and 6A^4^) and SP-A2 (1A^0^ and 1A^3^), the most frequently observed variants in the general population, were expressed from stably transfected Chinese Hamster Ovary (CHO) mammalian cells as previously described (60, 61). CHO-expressed SP-A variants were expressed, purified, and concentrated, and their purity assessed by silver staining. Similar preparations of 6A^2^, 6A^4^, 1A^0^ and 1A^3^ were used for this work (60, 61).

### SP-A binding assay (ELISA)

Polystyrene microtiter plates were coated with either 1 µg/mL recombinant SARS-CoV-2 spike protein produced in HEK293T cells (10549-CV-100, R&D Systems and Biotech, NE, MN, USA), while some plates were immobilized with 2.5 µg/mL WT RBD (SARS-CoV-2 inhibitor screening kit VANCOOB, R&D Systems) overnight at 4 °C in sodium carbonate buffer (pH 9.6). The S protein has mutations that ensure its prefusion conformation. After coating, the plates were washed four times with TBST (pH 7.4-7.6) and blocked with 3% BSA diluted in TBS buffer for 1:30 mins at room temperature (RT). Then we added a series of two-fold dilutions of individual purified recombinant human SP-A variants 6A^2^, 6A^4^, 1A^0^, or 1A^3^ (0-10 µg/mL, 100 µL/well) in TBST containing either 5 mM CaCl_2_ (Sigma-Aldrich, Saint Louis, MO, USA) and incubated at room temperature for 1 h. The wells were washed 4 times and incubated with SP-A IgG polyclonal antibody (1:1000) at room temperature for 1 h with slight shaking and SP-A-S protein or SP-A-RBD complexes were detected by adding HRP-conjugated Goat Anti-rabbit IgG (1:2000, Bio-rad, Hercules, USA) for an additional 1 h. The absorbance (450 nm) of individual wells was quantified using a spectrophotometer (Multiscan Ascent, Labsystem; Fisher Scientific, NH, USA). Experiments were carried out in triplicates from three independent experiments.

### SARS-CoV-2 (delta variant) infectivity assays in the presence of recombinant human SP-A genetic variants

To further assess the role of SP-A genetic variants (6A^2^ and 1A^0^) in SARS-CoV-2 infectivity, we pre-incubated SARS-CoV-2 (Delta) with individual SP-A gene-specific variants (0 and 10 μg/mL) for 1 h before inoculating confluent A549-ACE2 cells with the virus + protein mixture for 1 h at 37 °C (MOI = 0.05), unbound virus was washed, and fresh growth media added, and cells incubated at 37 °C for 24 h. Cells and culture media were collected and viral RNA, protein, and titer in cells and media were analyzed.

### SARS-CoV-2 (delta variant) binding, entry, and infectivity in the presence of human SP-A

Binding assays were performed by pre-incubating infectious SARS-CoV-2 (B.1.617.2) with human SP-A for 1 h before inoculating pre-chilled A549-ACE2 cells with virus/protein mixture at 4 °C for 2 h to allow virus binding to the cell surface. The cells were washed four times with cold PBS to remove unbound viral particles. Total RNA was isolated, and the amount of virus on cells was quantified by real-time quantitative PCR (RT-qPCR). For entry assays, after 2 h incubation of SP-A + virus mixture at 4 °C, the cells were washed, and fresh media was added and shifted to 37 °C for 1 h to allow virus entry into cells. Then the cells were washed and treated with proteinase K (1 mg/mL) to remove attached viral particles on the cell surface, and the amount of internalized viral particles was quantified by RT-qPCR. To further assess the role of SP-A in SARS-CoV-2 entry and infectivity, we pre-incubated the virus with SP-A (0 to 50 µg/mL) for 1 h before inoculating confluent A549-ACE2 cells with the virus + protein mixture for 2 h at 37 °C (MOI = 0.05). Unbound viral particles were washed, and fresh growth media was added and incubated at 37 °C. Cells and supernatant were collected at 4 and 24 hpi, and viral RNA, protein, and titer in cells and supernatant were analyzed. We also assessed the antiviral role of SP-A by pretreating A549-ACE2 with SP-A for 2 h before virus inoculation (MOI = 0.05) for another 1 h. Cells and media were collected at 24 hpi for further analyses.

### Real-time quantitative PCR

Total RNA was isolated from cell lysates using the Quick-RNA extraction miniprep kit (# R1055 Zymo Research, CA, USA) following the manufacturer’s instructions. RT-qPCR was performed using the AB StepOnePlus Detection System as previously described (56). The data were analyzed as fold change in CT values compared to SP-A untreated samples using the 2^-ΔΔCt^ method.

### Measurement of viral loads

Using RT-qPCR for viral genome copy number determination, total RNA was isolated from homogenized tissues using the Quick-RNA extraction miniprep kit (# R1055 Zymo Research, CA, USA) following the manufacturer’s instructions, and RNA concentration was determined by the nanodrop machine (Thermo Scientific). RT-qPCR was performed using the AB Step OnePlus Detection System (Applied Biosystems, Foster City, CA). and the one-step kit RT-PCR Master Mix Reagents (#64471423, Biorad). Reaction mixtures were prepared according to the manufacturer’s instructions. In brief, purified RNA was added into a 10 µl total volume of real-time PCR mix buffer. The one-step RT-qPCR was carried out through one cycle of reverse transcription at 55 °C for 10 mins followed by 40 cycles of amplification at 95 °C for 3 mins, 95 °C for 15 s, and 55 °C for 1 min as previously described (56, 62).

### Immunoblotting analysis

Cells were lysed using RIPA buffer (ThermoFisher Scientific, Rockford, IL) with a cocktail of protease and phosphatase inhibitors (Roche, Indianapolis, IN). Total protein in cell lysates was measured using the BCA protein assay kit (ThermoFisher Scientific). Total protein was resolved by SDS-PAGE on a 10% gel and transferred into PVDF membranes (Bio-Rad). The blots were blocked in a buffer containing 5% non-fat milk for 30 mins and incubated overnight at 4 °C with SARS-CoV-2 nucleocapsid protein antibody (NB100-56576, Novus Biological, CO, USA). As a loading control, blots were stripped and re-probed with GAPDH antibody. Subsequently, the membranes were incubated with goat anti-mouse IgG HRP-conjugated secondary antibody (Bio-Rad) and developed using ECL Western Blotting Substrate (ThermoFisher Scientific).

### Statistical analysis

All experimental data are presented as mean ± standard error and statistically analyzed using GraphPad Prism 8.0 (GraphPad Software, San Diego, CA, USA). Comparisons between two independent groups were performed using two-sided Student’s *t*-test or multiple groups using one-way ANOVA. Statistical comparisons were not performed unless a group contained at least 3 samples. Results were considered statistically significant when P<0.05.

## Results

### Human SP-A variants and sex differentially attenuate SARS-CoV-2-induced ALI in mice

**Figure 1:**
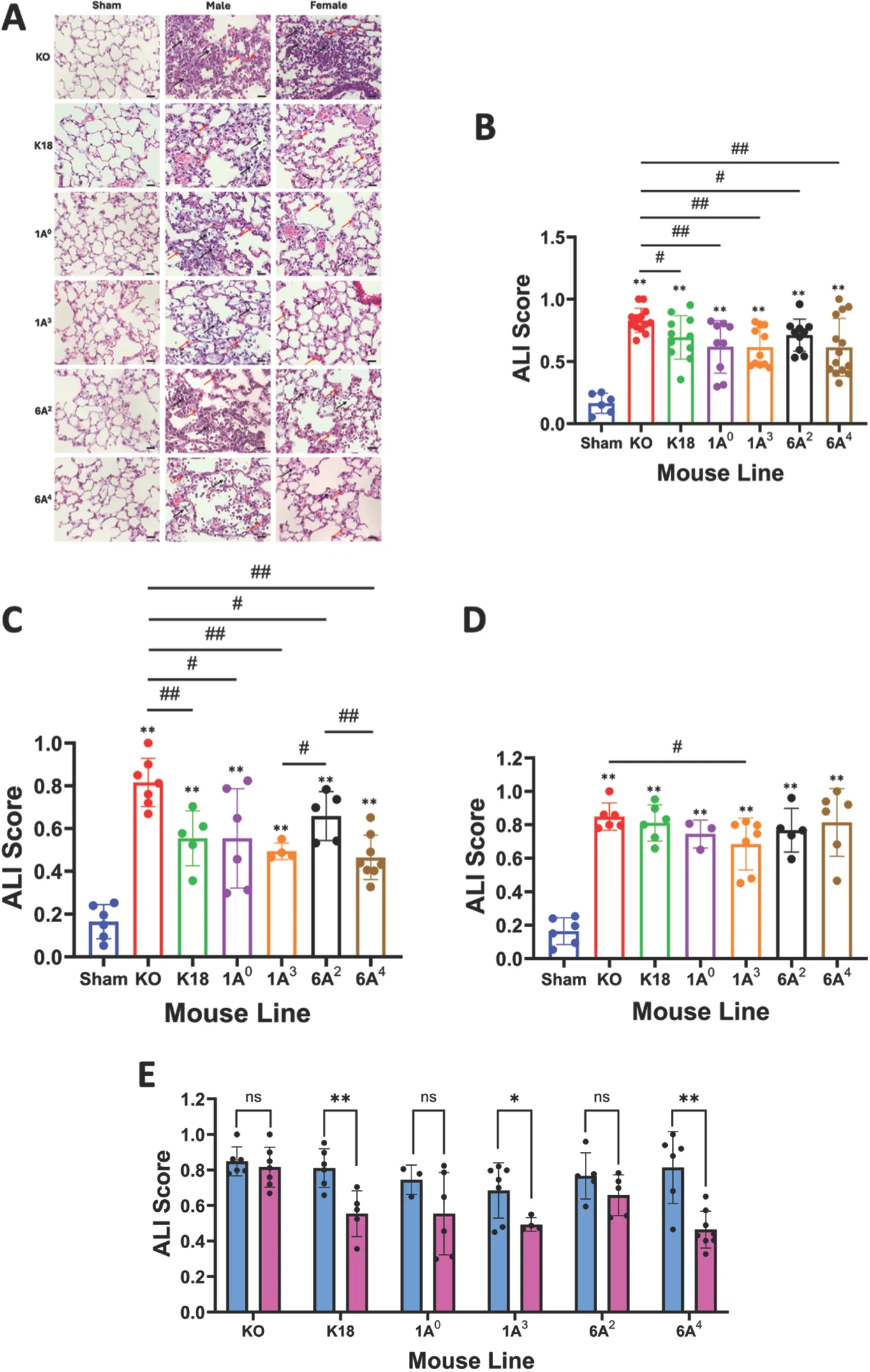
Effects of human SP-A variants and sex in lung injury of infected mice. **(A)** Representative histological sections of lungs from each mouse group on day 6 pi. SARS-CoV-2 induced a more severe lung damage in KO mice characterized by an infiltration of inflammatory cells in alveolar spaces and interstitium (black arrows), accumulation of proteinaceous debris, and edema leakage of protein-rich fluid in the alveolar spaces **(red arrows)** compared to K18 and SP-A hTG. Bars= 25µm, 400X magnification. **(B)** Quantified acute lung injury scores of each mouse group, ignoring sex. All infected groups showed significantly lower injury scores vs KO. Data are means ± SEM, significance was calculated using two-sided Student t-tests, **P < 0.01 (vs Sham); #<0.05, ##<0.01 (Between two infected groups; n = 6–14 mice/group). **(C)** Quantified acute lung injury scores of each mouse group after isolating only female samples. All infected groups showed significantly lower injury scores vs KO with 1A^3^ and 6A^4^ being the most attenuated. Data are means ± SEM, significance was calculated using two-sided Student t-tests, **P < 0.01 (vs Sham); #<0.05, ##<0.01 (Between two infected groups; n = 4–8 mice/group). **(D)** Quantified acute lung injury scores of each mouse group after isolating only male samples. Only 1A^3^ males had a lower lung injury score that reached significance vs. KO males. Data are means ± SEM, significance was calculated using two-sided Student t-tests, **P < 0.01 (vs Sham); #<0.05 (Between two infected groups; n = 3–7 mice/group). **(E)** Quantified acute lung injury scores compared between male and female mice of each infected group. K18, 1A^3^, and 6A^4^ males all demonstrated a more significant mean lung injury score vs their female counterparts. Data are means ± SEM, Blue = male mice, Pink = female mice, significance was calculated using two-sided Student t-tests, *P < 0.05, **P < 0.01, ns=no significance (male vs female within each infection group).

To decipher the protective role of SP-A genetic variants following SARS-CoV-2 infection, we assessed ALI variants on day 6 pi in four different SP-A hTG mice: 6A^2^, 6A^4^, 1A^0^, and 1A^3^ along with K18 and KO mice. ALI characteristics in the respective mouse lines were examined from lungs collected on day 6 post-infection (pi) by H&E and injury was quantified as recommended by the American Thoracic Society (57). In this study, we observed the presence of inflammatory cells in the alveolar space and interstitium (black arrows, **Figure 1A**), proteinaceous debris within the airways, and a thickening of alveolar walls resulting from a loss of alveolar integrity and fluid accumulation (red arrows, **Figure 1A**) in infected lungs. As expected, none of these characteristics were found in the lungs of sham mice (**Figure 1A**). After infection, we observed that all infected mouse groups had significantly higher ALI scores vs sham. When observing both male and female mice combined, the markers of severe ALI were significantly more prominent in KO mice compared to all hTG mouse groups (degree of severity of ALI score was as follows: 6A^2^>1A^0^>K18>1A^3^>6A^4^) (**Figure 1B**). We did not see a significant difference between the various transgenic groups when males and females were combined (**Figure 1B**).

When examining only female mice from each group, we observed that all infected mouse groups had significantly lower ALI scores vs KO and significantly higher scores vs sham (**Figure 1C**). The degree of severity of ALI in female only mouse groups was as follows: 6A^2^>K18>1A^0^>1A^3^>6A^4^ (**Figure 1C**). Additionally, 1A^3^ and 6A^4^ females had significantly lower ALI scores when compared vs female 6A^2^ mice, largely due to a lower average level of neutrophil infiltration observed in each sample (**Figure 1C**). When examining only male mice from each group, we observed that all infected mouse groups had significantly higher scores vs sham but only 1A^3^ males had a significantly lower ALI score vs KO males, also primarily due to differences in neutrophil presence (**Figure 1D**). When comparing male vs female mice within each mouse group, we observed that female K18, 1A^3^, and 6A^4^ mice exhibited lower ALI scores vs their male counterparts and observed no difference in KO, 1A^0^, or 6A^2^ mice. (**Figure 1E**). This indicates that both female sex and SP-A protect against severe ALI following SARS-CoV-2 infection, largely by modulating local neutrophil infiltration, but its host defense function differs based on the unique SP-A variant expressed with 1A^3^ and 6A^4^ females being the most protected.

### Human SP-A variants and sex differentially attenuate SARS-CoV-2-induced kidney injury

**Figure 2:**
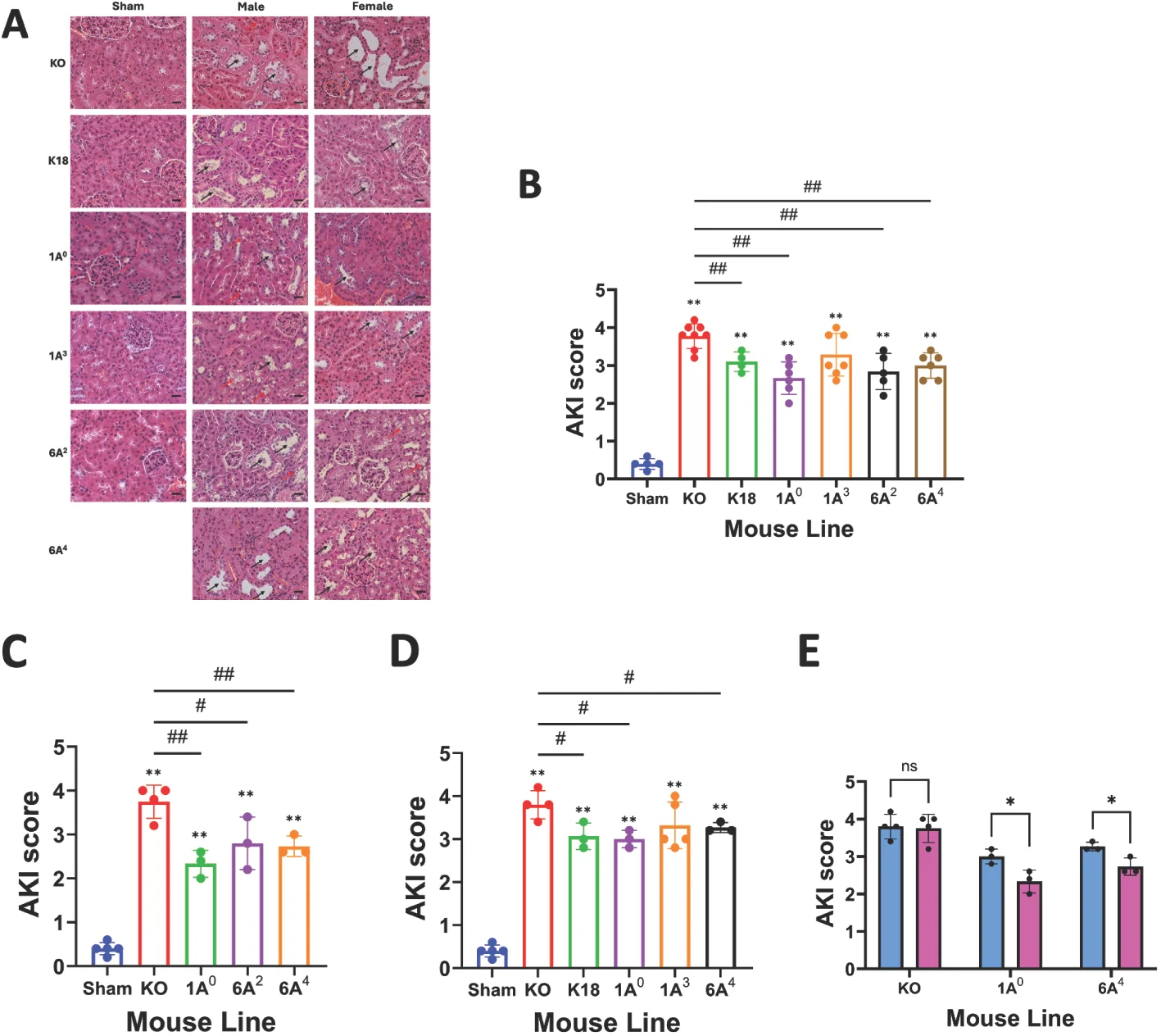
Effects of human SP-A variants and sex in kidney injury of infected mice. **(A)** Representative histological sections of kidneys from each mouse group on day 6 pi. SARS-CoV-2 induced more severe kidney damage in KO mice characterized by SARS-CoV-2-induced acute kidney injury **(AKI)** was characterized by the presence of vacuolar degeneration of tubular cells (red arrows and loss of brush border with dilatation of tubular lumen and cast formation in all infected mice (black arrows). Bars= 25µm, 400X magnification. **(B)** Quantitative analysis of acute kidney injury of each mouse group, ignoring sex. All infected groups less 1A^3^ showed a significantly lower renal injury vs SP-A deficient **(KO)** mice. Data are means ± SEM, significance was calculated using two-sided Student t-tests, **P < 0.01 (vs Sham); #<0.05, ##<0.01 (Between two infected groups; n = 3–8 mice/group, bars= 25µm, 400X magnification). **(C)** Quantified acute renal injury scores of each mouse group after isolating only female samples with sufficient sample size for statistical analysis. All infected groups showed significantly lower injury scores vs KO. Data are means ± SEM, significance was calculated using two-sided Student t-tests, **P < 0.01 (vs Sham); #<0.05, ##<0.01 (Between two infected groups; n = 3–4 mice/group). **(D)** Quantified acute renal injury scores of each mouse group after isolating only male samples. All infected groups showed significantly lower injury scores vs KO except for 1A^3^. Data are means ± SEM, significance was calculated using two-sided Student t-tests, **P < 0.01 (vs Sham); #<0.05 (Between two infected groups; n = 3–5 mice/group). **(E)** Quantified acute renal injury scores compared between male and female mice of each infected group. 1A^0^ and 6A^4^ males all demonstrated a more significant mean kidney injury score vs their female counterparts. Data are means ± SEM, Blue = male mice, Pink = female mice, significance was calculated using two-sided Student t-tests, *P < 0.05, ns=no significance (male vs female within each infection group).

Given the increasing reports of acute kidney injury (AKI) post-SARS-CoV-2 infection, we assessed injury in the kidneys of mice carrying individual human SP-A variants on day 6 pi. Normal kidney architecture was observed in the sham group, suggesting that SP-A deficiency did not alter kidney development and structure (**Figure 2A**). SARS-CoV-2-induced AKI was characterized by the presence of vacuolar degeneration of tubular cells (red arrows, **Figure 2A**) and loss of brush border with dilatation of tubular lumen and cast formation in all infected mice (black arrows, **Figure 2A**), although with different severities. After infection, we observed that all infected mouse groups had significantly higher AKI scores vs sham (**Figure 2B**). When observing both male and female mice combined, the markers of severe AKI were significantly more prominent in KO mice compared to all hTG mouse groups except for 1A^3^ (degree of severity of AKI score was as follows: K18>6A^4^>6A^2^>1A^0^) (**Figure 2B**). We did not see a significant difference between the various transgenic groups when males and females were combined (**Figure 2B**).

When examining only female mice from each group with sufficient sample size (6A^2^, 6A^4^, and 1A^0^), we observed that all infected mouse groups had significantly lower AKI scores vs KO and significantly higher scores vs sham (K18 and 1A^3^ groups did not have sufficient sample size for female calculations) (**Figure 2C**). This was based on a lower percentage of tissue in groups with significance demonstrating signs of injury. The degree of severity of AKI in female-only mouse groups was as follows: 6A^2^>6A^4^>1A^0^ (**Figure 2C**). There was no significant difference in AKI between female hTG groups (**Figure 2C**). When examining only male mice from each group with sufficient sample size (K18, 6A^4^, 1A^3^, 1A^0^), we observed that K18, 6A^4^, and 1A^0^ males had significantly lower AKI scores vs KO males and that all infected mouse groups significantly higher scores vs sham (1A^3^ was not significant vs KO and 6A^2^ did not have sufficient sample size for male calculations) (**Figure 2D**). The degree of severity of AKI in male-only mouse groups was as follows: 6A^4^>K18>1A^0^ (**Figure 2D**). There was no significant difference in AKI between male hTG groups (**Figure 2D**). When comparing male vs female mice within each mouse group, we observed that female 1A^0^ and 6A^4^ mice exhibited lower AKI scores vs their male counterparts and again no difference in KO mice (**Figure 2E**). These female groups were more often characterized by the presence of individual tubular cell vacuolar degeneration and death and fewer tubular casts as compared to male samples, who more often demonstrated more major signs of kidney injury (lumen dilation, loss of brush border, and large cast formation). This indicates that both female sex and SP-A protect against severe AKI following SARS-CoV-2 infection, but its host defense function at the kidney differs based on the unique SP-A variant expressed.

**Figure 3:**
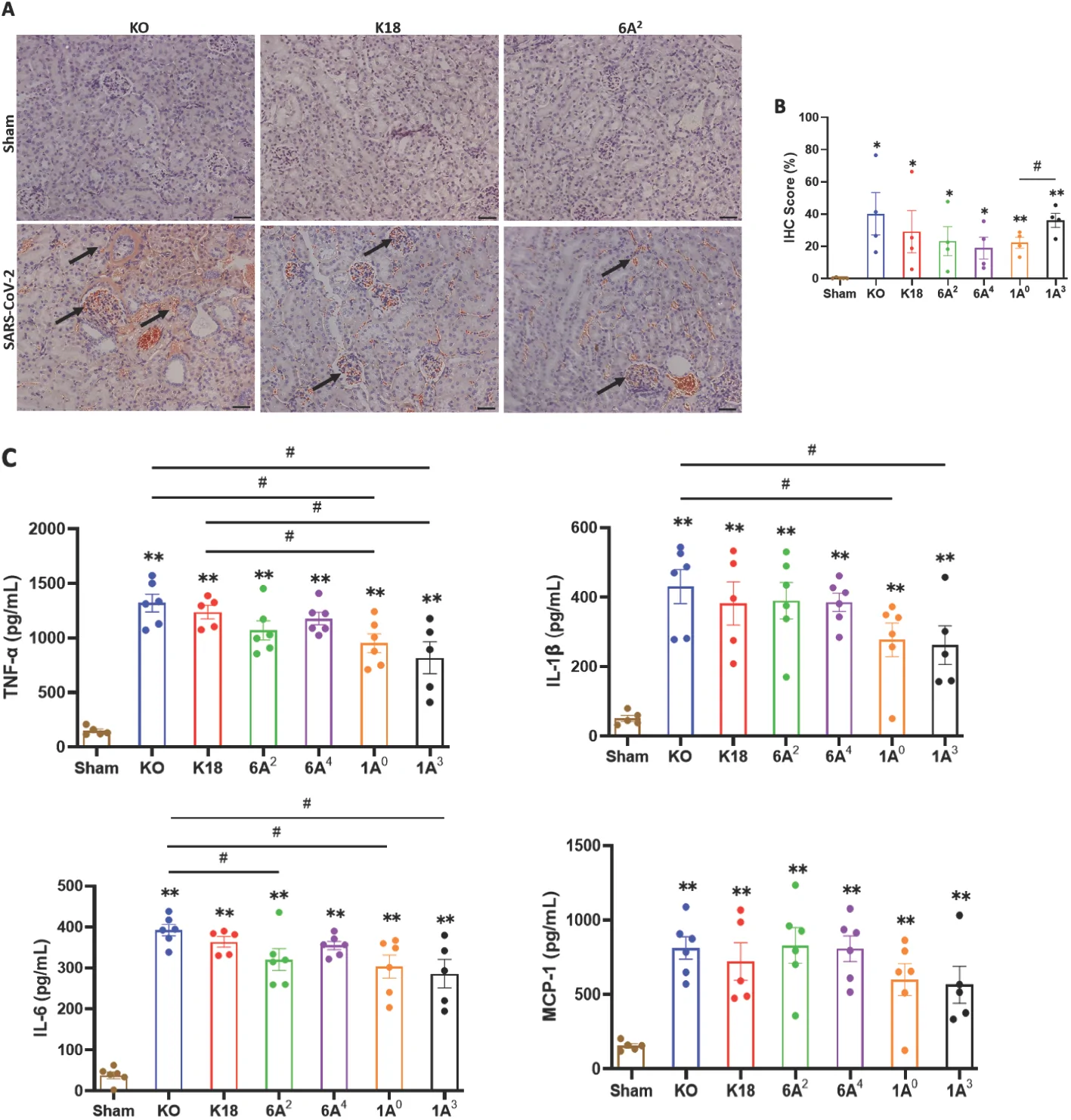
Human SP-A variants influence viral burden and inflammation cytokine levels in the kidney of infected mice. **(A)** Representative IHC staining for SARS-CoV-2 nucleocapsid protein (NP) distribution in the kidney following intranasal inoculation of SARS-CoV-2 on day 6 PI shows remarkable infection of cytoplasmic and perinuclear localization of N SARS-CoV-2 NP in renal tubular cells in all infected mouse groups compared to sham. **(B)** Semi-quantitative analysis revealed a significant presence of SARS-CoV-2 NP in all infected mouse groups compared to sham mice. Data are means ± SEM, **P < 0.01 (vs Sham); #<0.05 (Between two infected groups) (n = 4–7 mice/group, bars= 50µm, 200X magnification). **(C)** Levels of TNF-α, IL-6, IL-1β, and MCP-1 were measured by ELISA. Infected KO mice generally had higher levels of these mediators (except MCP-1) in kidneys than in other lines. Data are means ± SEM, **P < 0.01 (vs Sham); #<0.05 (Between two infected groups; n = 4–7 mice/group, bars= 50µm, 200X magnification).

We reasoned that the differences in severity may have been mediated by the differential capacity of SP-A variants to inhibit viral infectivity. Thus, we quantified viral N protein titers in the kidney from each mouse group and found that viral load trended higher in KO mice and was significantly higher in 1A^3^ relative to 1A^0^ mice (**Figures 3A, B**). Moreover, all infected mouse groups were positive for viral N protein with 6A^4^ (more severe disease) having the lowest viral burden. To further delineate the reason for the differential pathological differences in mouse kidneys, we measured the levels of inflammatory cytokines from kidney homogenates. Strikingly, significantly reduced expressions of TNF-α, IL-1β, and IL-6 were observed in SP-A2 (1A^0^ and 1A^3^) relative to KO mice (**Figure 3C**). The levels of these cytokines were relatively higher in 6A^4^ mice (more severe AKI) compared to 1A^0^ (moderate AKI).

### Human SP-A variants differentially attenuate SARS-CoV-2-induced intestinal injury

**Figure 4:**
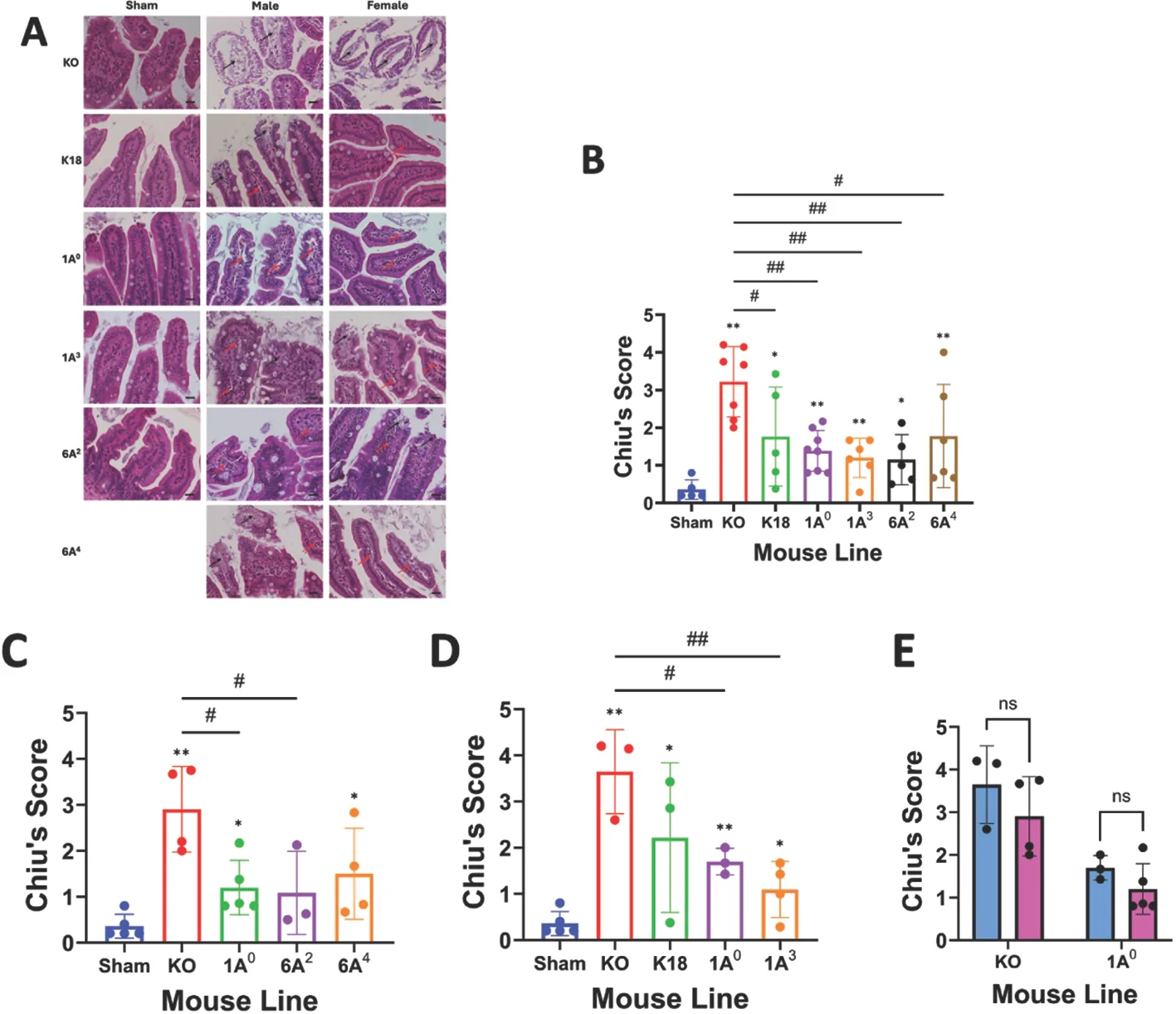
Effects of human SP-A variants and sex in intestine injury of infected mice. **(A)** Representative histological sections of small intestines from each mouse group on day 6 pi. SARS-CoV-2 induced more severe intestinal damage in KO mice characterized by the development of subepithelial space, capillary congestion, and epithelial lifting (red arrows) and denudation of villi and exposure and digestion of lamina propria (black arrows). Bars= 25µm, 400X magnification. **(B)** Quantitative analysis of acute intestinal injury of each mouse group, ignoring sex using Chiu’s scoring system. All infected groups showed a significantly lower Chiu’s score vs SP-A deficient (KO) mice. Data are means ± SEM, significance was calculated using two-sided Student t-tests, *P < 0.05, **P < 0.01 (vs Sham); #<0.05, ##<0.01 (Between two infected groups; n = 3–8 mice/group, bars= 25µm, 400X magnification). **(C)** Quantified acute intestinal injury scores of each mouse group after isolating only female samples with sufficient sample size for statistical analysis. Infected 1A^0^ and 6A^2^ male mice showed significantly lower injury scores vs their male SP-A KO counterparts. Data are means ± SEM, significance was calculated using two-sided Student t-tests, **P < 0.01 (vs Sham); #<0.05 (Between two infected groups; n = 3–5 mice/group). **(D)** Quantified acute intestinal injury scores of each mouse group after isolating only male samples. Infected 1A^0^ and 1A^3^ females showed significantly lower injury scores vs KO. Data are means ± SEM, significance was calculated using two-sided Student t-tests, **P < 0.01 (vs Sham); #<0.05, ##<0.01 (Between two infected groups; n = 3–4 mice/group). **(E)** Quantified acute intestinal injury scores compared between male and female mice of each infected group with sufficient sample sizes for statistical analysis. Neither infected KO nor 1A^0^ groups demonstrated a sex related difference in intestinal injury score. Data are means ± SEM, Blue = male mice, Pink = female mice, significance was calculated using two-sided Student t-tests, ns=no significance (male vs female within each infection group).

To examine the degree of intestinal injury in mice carrying the respective human SP-A variants, we measured Chiu’s score from each mouse group as a correlate of disease severity. Normal intestinal architecture was observed in the sham group, suggesting that SP-A deficiency did not alter intestinal development and structure (**Figure 4A**). SARS-CoV-2-induced acute intestinal injury as quantified by Chiu’s score was characterized by differing combinations of the following based on severity including the development of subepithelial space, capillary congestion, and epithelial lifting (red arrows, **Figure 4A**) and denudation of villi and exposure and digestion of lamina propria (black arrows, **Figure 4A**). When comparing acute intestinal injury scores between different mouse groups ignoring sex, Chiu’s scoring revealed that all infected transgenic groups exhibited significantly more injury vs infected KO mice 6A^4^>K18>1A^0^>1A^3^>6A^2^ and that only infected KO, 1A^0^, and 1A^3^ mouse groups were significantly different from our sham group (**Figure 4B**). We did not see a significant difference in Chiu’s scores between the various transgenic groups when males and females were combined (**Figure 4B**).

When examining only female mice from each group with sufficient sample size (KO, 1A^0^, 6A^2^, 6A^4^), we observed a significant difference in Chiu’s scores in 1A^0^ and 6A^2^ mouse groups vs KO and only a significant difference vs sham in the female KO group (**Figure 4C**). When examining only male mice from each group with sufficient sample size (KO, K18, 1A^0^, 1A^3^), we observed a significant difference in intestinal injury in 1A^0^ and 1A^3^ (**Figure 4D**). The K18 male group and female 6A^4^ groups showed more severe breakdown of the villi with significant denudation of more than just tips and even recurrent examples of digestion of the lamina propria. We only saw a significant difference when comparing vs the sham mouse group in the KO and 1A^0^ mouse groups (**Figure 4D**). Regarding comparison between male and female mice with large enough group sizes for statistical comparison (KO and 1A^0^), there was no significance observed (**Figure 4E**). These findings indicate that SP-A variants protected from more severe SARS-CoV-2-induced intestinal injury. Our findings based on our limited quantity of statistically analyzable groups could not conclude that female plays a role in modulating severity of SARS-CoV-2-induced acute intestinal injury.

### Human SP-A variants differentially bind to SARS-CoV-2 S protein and RBD

**Figure 5:**
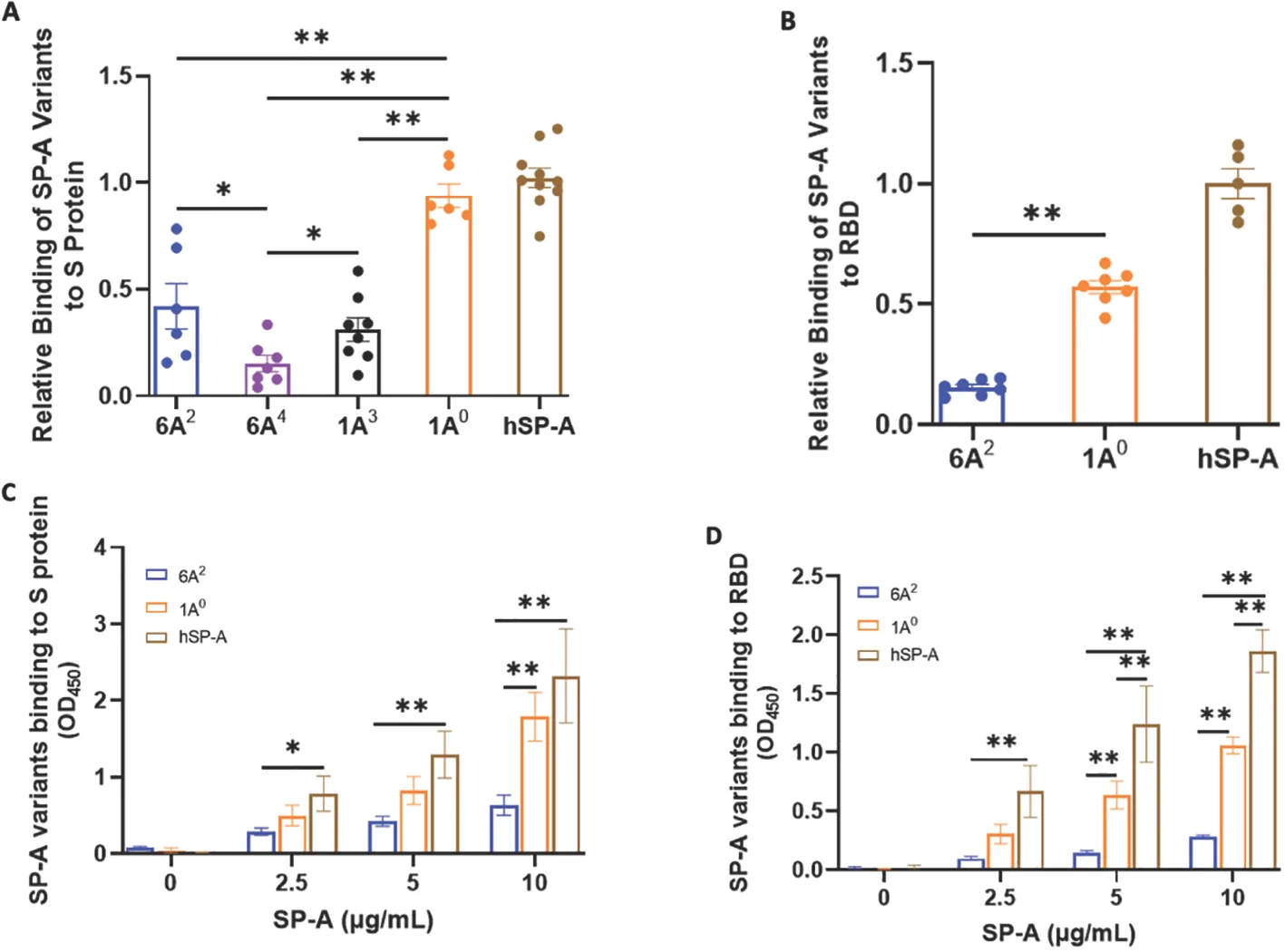
Human SP-A variants differentially bind to SARS-CoV-2 S protein and RBD. **(A, B)** Purified S protein and RBD were immobilized on ELISA plates followed by incubation with 10 μg/ml of each variant (6A^2^, 6A^4^, 1A^0^ or 1A^3^). The data showed a variant-dependent binding to SARS-CoV-2 S protein, with 1A^0^ having the most interaction with S protein while 6A^4^ had the least interaction of all the variants with S protein. In **(B)**, a more potent binding to immobilized SARS-CoV-2 RBD relative to native human SP-A was observed in the presence of 1A^0^ than 6A^2^. The ODs were normalized to those observed in the presence of native human SP-A. **(C-D)** A SP-A dose-dependent binding analysis. Purified S protein **(C)** and RBD **(D)** were immobilized on ELISA plates followed by incubation with increasing concentrations of 6A^2^ and 1A^0^ (0-10 μg/ml). We observed significant binding to S protein and RBD starting at 10 and 5 μg/ml, respectively. The data are presented as mean ± SE from at least 3 independent experiments. (*P<0.05; **P<0.01 reflect the levels of statistical significance between the variants).

Given that we observed differences in the degree of ALI severity and mortality in our mouse lines, we further wanted to elucidate whether the differences in severity were because of differential antiviral effects of individual human SP-A variants in response to SARS-CoV-2 infection using a human lung epithelial cell line. In our previous study, we observed that native human SP-A binds to the structural proteins of SARS-CoV-2. Here, using *in vitro*-expressed variants derived from SP-A1 (6A^2^ and 6A^4^) and SP-A2 (1A^0^ and 1A^3^), we determined their potential interaction with SARS-CoV-2 S protein and RBD. We observed that all the variants (except 6A^4^) at 10 µg/mL significantly bound to the S protein (**Figure 5A**) with the most binding observed in the 1A^0^ variant (1A^0^>6A^2^>1A^3^>6A^4^). Further experiments were performed using only the 6A^2^ and 1A^0^ variants because they were observed to have the highest S protein binding and they are the most frequent SP-A genetic variants in the general population (61, 63). We observed a significantly higher binding (>40%) to SARS-CoV-2 RBD with the 1A^0^ variant compared to the 6A^2^ variant at 10 µg/mL (**Figure 5B**). As shown in **Figures 5C, D**, using three different concentrations of 6A^2^ and 1A^0^, we further observed a dose-dependent increase in the binding of both variants to SARS-CoV-2 S protein and RBD. Variant 1A^0^ bound more significantly to the S protein and RBD, exhibiting approximately 3 times more interaction with S protein than variant 6A^2^ at 10 µg/mL dose (**Figure 5C**, mean ODs: 1A^0^ = 1.79, 6A^2^ = 0.63). As observed in **Figure 5D**, 1A^0^ variant more strongly recognized RBD starting at 5 µg/ml relative to the 6A^2^ variant. These findings suggest that there are differences in the capacity of SP-A1 and SP-A2 genetic variants to bind to SARS-CoV-2 proteins and that the 1A^0^ variant may have more functional capacity in response to SARS-CoV-2 infection than the other variants.

### Human SP-A variants differentially inhibit SARS-CoV-2 infectivity in A549-ACE2 cells

**Figure 6:**
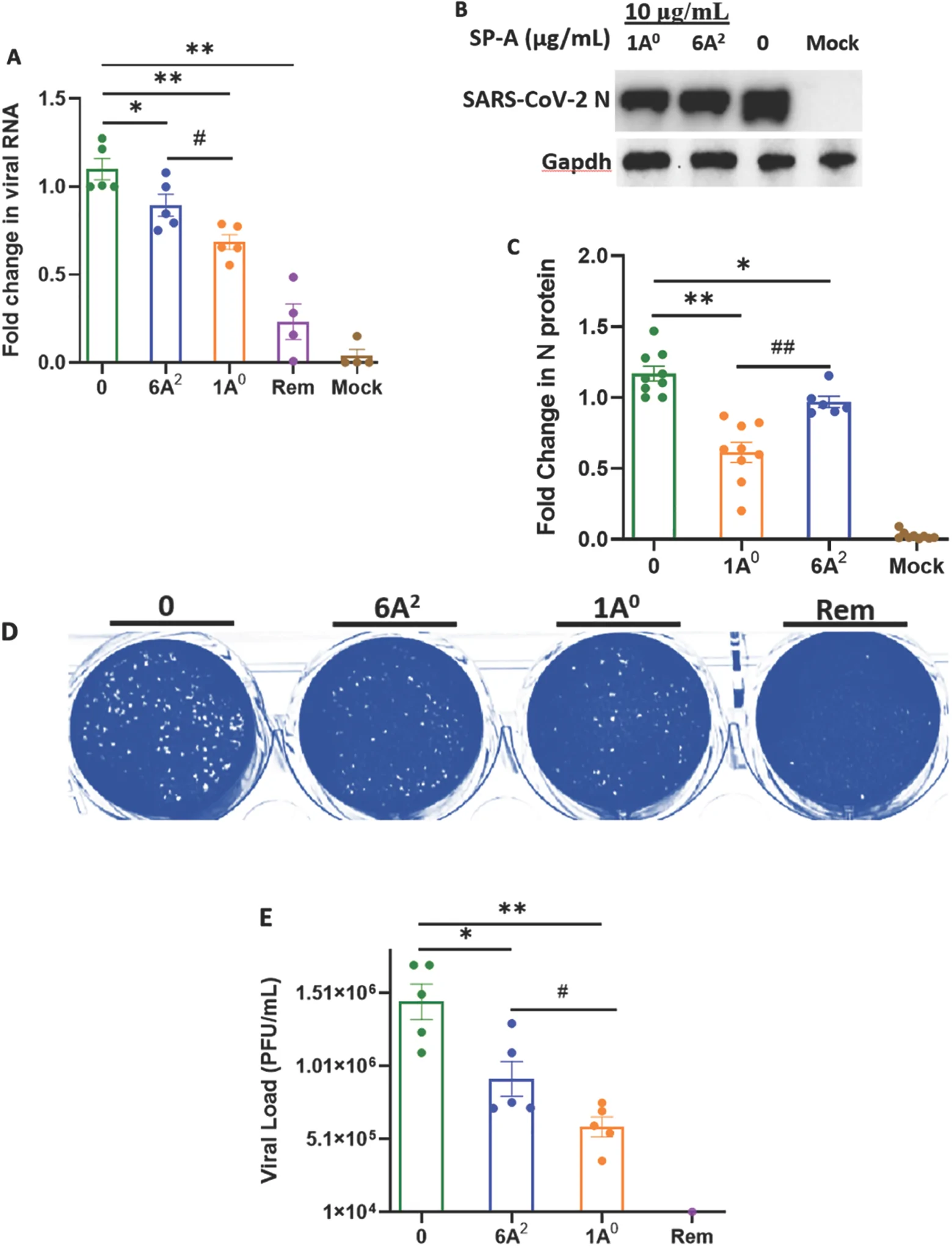
Human SP-A variants inhibits SARS-CoV-2 infectivity in A549-ACE2 cells. **(A)** SARS-CoV-2 was pre-incubated with 10 µg/ml of 1A^0^ and 6A^2^ before inoculating confluent A549-ACE2 cells. Cell culture media and cells were collected and used for RT-qPCR **(A)**, western blot (B&C), and plaque assays (D&E). **(A)** A significant decrease in viral RNA levels in cells was observed in the presence of both variants (1A^0^>6A^2^). 1 µM Remdesivir was used as a positive control. **(B)** Western blots of SARS-CoV-2 N expressions in cell lysates revealed reduced NP in cells in the presence of both variants compared to 0 µg/ml **(C)** Relative viral N protein levels in A549-ACE2 cell lysate was normalized to GAPDH and revealed significantly reduced NP levels in cells with 1A^0^ relative to 6A^2^. **(D)** Visible plaques on Vero E6 cells infected with or without (10 μg/ml) 1A^0^ or 6A^2^. **(E)** Significantly reduced virus titer with both 6A^2^ and 1A^0^ (1A^0^>6A^2^). Values represent mean ± S.E. *P<0.05, **P<0.01, compared to the control sample (0 μg/ml) and #<0.05, ##<0.01 when compared between the variants (n= 5).

Given the observed interaction between SP-A variants and SARS-CoV-2 RBD, necessary for host cell entry, we further examined the impact on viral infectivity in a susceptible cell line. We inoculated A549-ACE2 cells with SARS-CoV-2 (Delta variant) preincubated with or without 10 µg/ml of either 6A^2^ or 1A^0^. The results showed a significant decrease in viral RNA levels in cells in the presence of both variants especially in cells incubated with 1A^0^ (**Figure 6A**). This finding was further confirmed by immunoblotting where we observed a significant decrease in the level of viral N protein in the presence of both variants, a decrease that was even more significant in cells inoculated with the virus and the 1A^0^ mixture (**Figures 6B, C**). 10 µg/ml of both variants resulted in significant decrease in infectious titer and an approximately one-half log reduction in viral titer in the presence of 1A^0^ variant (**Figures 6D, E**). Taken together, these results suggest that human SP-A variants (1A^0^ and 6A^2^) tested in this study can bind to SARS-CoV-2 S protein and inhibit infectivity (albeit differentially) in lung epithelial cells. Variant 1A^0^ more potently interacts with SARS-CoV-2 and inhibits viral infectivity in susceptible cells as compared to variant (6A^2^).

## Discussion

While COVID-19 manifestations are largely heterogenous and a significant number of people remain asymptomatic post-infection; studies have shown that some infected individuals develop acute and persistent multiple organ dysfunctions (MOD) affecting the pulmonary, cardiac, gastrointestinal, renal, and nervous systems post SARS-CoV-2 infection (64–66). MOD has been linked with broad expression of hACE2 in several organs, active viral replication, and high levels of local and systemic inflammatory cytokines (67–70). The importance of human SP-A against the diverse microbes encountered throughout human evolution has necessitated its robust genetic variation to effectively protect the host from emerging and re-emerging pathogens (71). These genetic changes have been mapped to the functional domains of SP-A that play crucial roles against invading pathogens by recognizing and neutralizing infection and modulating inflammation via specific host-cell receptor interactions. In this study, using SARS-CoV-2 susceptible male and female mice expressing single gene variants of SP-A1 or SP-A2, we elucidated the mucosal innate immune roles of each variant and sex in modulating SARS-CoV-2-induced mortality and severity of acute lung, kidney, and intestinal injury. Sex differences in COVID-19 severity are well established but the interaction between sex and SP-A variant in modulating multi-organ injury has not yet been characterized (72, 73). We examined sex as a co-variable alongside SP-A variant to characterize their relationship *in vivo*. We further assessed the binding capacity of the individual variants to SARS-CoV-2 structural proteins and the impact on infectivity *in vitro*.

### Sex differences and SP-A variant in SARS-CoV-2 induced multi organ injury

The findings from this *in vivo* study demonstrate that variants of SP-A1 and SP-A2 and biological sex interact to modulate SARS-CoV-2 induced multi-organ injury in a tissue-specific manner. Despite the well-established male predominance in COVID-19 hospitalizations, ICU admission, and deaths (72, 73), the underlying molecular mechanism for this sex disparity has yet to be fully uncovered. There is significant evidence that estrogens promote effective immune responses and androgens do the opposite (74–77). Several immune-cell-related genes on the X chromosome further contribute to female immune advantage (78, 79). This study found that KO mice showed no sex difference in lung or kidney injury severity scoring with a sex-based protection observed in the presence of specific SP-A variants. This suggests that these hormonal and chromosomal variants may interact with SP-A as a biological intermediary when modulating MOD. The mechanisms underlying the interaction between biological sex and SP-A variants are likely multifactorial. Sex hormones may alter the epithelial and immune-cell environment in which SP-A functions rather than interact directly with the SP-A molecule. Androgen signaling can increase the expression of SARS-CoV-2 entry-associated factors, including ACE2 and TMPRSS2, while testosterone may suppress selected antiviral and innate immune responses (46–48). Conversely, estrogen has been reported to reduce ACE2 expression in some tissues and to modulate inflammatory, interferon and immune-cell responses (54). These effects may influence viral burden, epithelial injury and the activation states of neutrophils and macrophages, which are major cellular targets of SP-A immunomodulatory activity. Because SP-A regulates pathogen binding, opsonization, phagocytosis and inflammatory signaling through multiple activating and inhibitory cell-surface receptors, male and female immune cells may respond differently to the same SP-A variant (71, 80, 81). Thus, SP-A may function as an interface through which sex-dependent epithelial and immune-cell programs modify the outcome of infection. The variant-specific nature of our findings is also important. Female protection was not observed uniformly among all SP-A-expressing mouse lines but was most evident with 1A³ and 6A^4^ in the lung and with 1A^0^ and 6A^4^ in the kidney. Structural and biochemical differences among SP-A1 and SP-A2 variants, including differences in oligomerization, glycosylation, microbial recognition and immune-cell signaling, may determine how effectively each variant functions within a sex-dependent hormonal and immunological environment. However, the present study establishes a phenotypic interaction between sex and SP-A genotype but does not demonstrate that estrogen, progesterone or androgen receptors directly regulate SP-A expression or signaling. Such mechanisms require direct experimental investigation in the future.

Prior works have shown that *SFTPA1* and *SFTPA2* variants differ by sex in their ability to regulate immune function, gene expression/signaling pathways of alveolar macrophages in the lung and survival under infection or inflammatory challenge (81, 82), and that alveolar macrophage responses to exogenous SP-A1 and SP-A2 are sex-dependent, with females more responsive to SP-A1 and males to SP-A2 (83). This study further examined these observations under the context of COVID-19 induced MOD and demonstrated that the sex-SP-A interaction is both variant and organ specific.

### SP-A variants 1A^3^ and 6A^4^ and female sex most attenuate acute lung injury

Post COVID-19 infection, female sex combined along with the 1A^3^ and 6A^4^ SP-A variants conferred the greatest attenuation of ALI, while only 1A^3^ reached significance relative to KO among male mice. This variant-specific effect suggests that not all SP-A alleles equally interact with the sex-based immune advantage. The structural differences between SP-A1 and SP-A2 variants likely factors into these variant-specific differences and may interact differently with sex hormone signaling pathways (60, 84). Given that a small group of individuals carrying 6A^4^ and 1A^3^ alleles appear highly protected from severe lung damage (61, 63), SP-A genotyping for patients may have potential as a pulmonary risk stratification tool in COVID-19. Based on the findings of this *in vivo* study, this is especially valid when considering SP-A variant alongside the patient’s biological sex. Documented prior observations of better lung function and mechanics in 1A^3^ mice as compared to 1A^0^ post-bacterial infection is further evidence of the importance of SP-A variant in stratifying risk (85).

### SP-A variants 1A^0^ and 6A^4^ and female sex most attenuate acute kidney injury

Large multicenter GWAS studies have found an increased risk of critical COVID-19 illness to be associated with reduced glomerular filtration rate and low kidney function (16, 17). Given that AKI is a frequent complication in COVID-19 that has been associated with fatal disease outcomes, we therefore examined the role of SP-A in modulating AKI severity following infection (69). The renal findings from this study reinforce the sex-specific role of SP-A variants while revealing important organ-specific divergence. In this *in vivo* study, female 1A^0^ and 6A^4^ mice exhibited the greatest renal protection against COVID-19 infection (as compared to female 1A^3^ and 6A^4^ mice for lung protection). This difference suggests that the function of each SP-A variant is not identical over different tissue environments, likely due to differences in local receptor expression or inflammatory signaling. This is consistent with prior observation that SP-A deficiency results in profound inflammation and severe renal fibrosis in animals (86). Direct viral cytopathic effect alongside robust systemic and local cytokine levels has been associated with severe renal dysfunction in patients (67, 87, 88). Reduced levels of SP-A and SP-D are frequently found in women with recurrent urinary tract infections (UTIs) (89) while the absence of SP-A and SP-D resulted in higher bacterial burden and increased levels of inflammatory gene mediators, suggestive of the potential role of collectins in maintaining kidney health (90). Our group previously observed expressions of SP-A1 and SP-A2 genetic variants in hTG mice kidneys and speculated their potential immunomodulatory roles in response to infection (91). Notably, SP-D, another closely related collectin, alleviated AKI severity in an animal model of pneumonia by attenuating apoptosis and inflammatory gene expressions (92).

Herein, we assessed viral load and levels of inflammatory mediators as correlates of severe kidney injury and observed differential viral burden among our hTG mice. The higher viral N protein in 1A^3^ mice (compared to 1A^0^ mice in **Figure 3B**) supports previous reports of a link between elevated viral load and severe COVID-19 AKI and is consistent with this study’s non-sex-based *in vivo* results (87). In addition, inflammatory cytokines were significantly reduced in 1A^0^ and 6A^2^ mice (more attenuated AKI) compared to 1A^3^ and 6A^4^ mice and consistent with the contribution of enhanced tissue cytokine expression and AKI severity (67, 68). It was previously demonstrated that SP-A binds and activates SIRP-α in renal podocytes resulting in proteinuria (93). Whether SP-A genetic variants directly interact with signaling molecules in renal tubular cells to modulate inflammation is yet to be demonstrated. Furthermore, the role of the variants in attenuating death pathways in renal cells should be explored further as it could unravel the mechanism of reduced AKI observed in most SP-A hTG lines and the contribution of each SP-A variant to the differences in nephropathy observed in COVID-19 patients (69).

### SP-A variants alone attenuate acute intestinal injury

According to a recent study, SARS-CoV-2 infected individuals are more than 50% more likely to develop ulcers in their stomach lining and intestines, suffer from irritable bowel syndrome, and digestive problems such as constipation, diarrhea, abdominal pain, vomiting, and bloating (94). Studies have reported gastrointestinal complications in COVID-19 patients with mild and severe disease (95–97). It has been shown that profound intestinal damage results from viral replication in ACE2-expressing enterocytes, influx of inflammatory mediators, and dysfunctions in gut immunological barriers (98–101). Since SP-A expression in the intestines was first described, a few studies have demonstrated its role in preventing GI graft vs host disease (GVHD), attenuating TLR-4-mediated inflammatory cytokine expression in mice intestinal ileum while SP-D was shown to be protective against inflammatory bowel disease (IBD) (102–104). The intestinal findings from this *in vivo* study presents a distinct divergence from the discussed lung and renal results: SP-A variants attenuated intestinal injury, but sex did not emerge as a significant modulating factor. This sharp contrast from the lung and kidney results raises the possibility that intestinal SP-A protection operates through a different, sex-independent mechanism. This implies that SP-A expression or their functions differ between different tissue compartments. The variance of biological female sex in modulating acute intestinal versus lung and kidney injury in our model may reflect a gap in the understanding of sex and surfactant protein in modulating MOD and warrants further investigation.

### Differential interaction of SP-A variants and SARS-CoV-2 S protein

To determine if the observed differences in ALI severity in the respective mouse lines was due to differences in the antiviral function of SP-A genetic variants, we pretreated infectious SARS-CoV-2 with individual SP-A variants before inoculating susceptible cells *in vitro*. Before this, we measured each SP-A variant’s ability to bind viral structural proteins. The variants were expressed in a mammalian cell expression system to maintain the glycosylation patterns observed in native human SP-A. For the first time, we demonstrate that individual human SP-A genetic variants can bind to SARS-CoV-2 S protein and RBD differentially. We further observed that SP-A2 (1A^0^) had the most interaction with viral structural proteins closely followed by SP-A2 (6A^2^). The biological significance of the differential S protein recognition was highlighted by the significantly reduced viral burden in lung epithelial cells infected with SARS-CoV-2 in the presence of 1A^0^ compared to 6A^2^. Thus, 1A^0^ can bind more to the virus and block subsequent binding and entry events in susceptible cells as was previously observed with native human SP-A in the context of SARS-CoV-2, HIV-1, and IAV infections (56, 105, 106). This finding is interesting due to *in vitro* evidence of the mucosal innate immune roles of SP-A genetic variants against SARS-CoV-2 infection. However, our *in vitro* data deviates from the similar hemagglutinating activity observed with 1A^0^ and 6A^2^ variants post-IAV infection (30). But, our data aligns with previous reports of enhanced bacterial phagocytosis and glycan binding with SP-A2 genetic variants compared to SP-A1 variants (29, 107).

**Figure 7:**
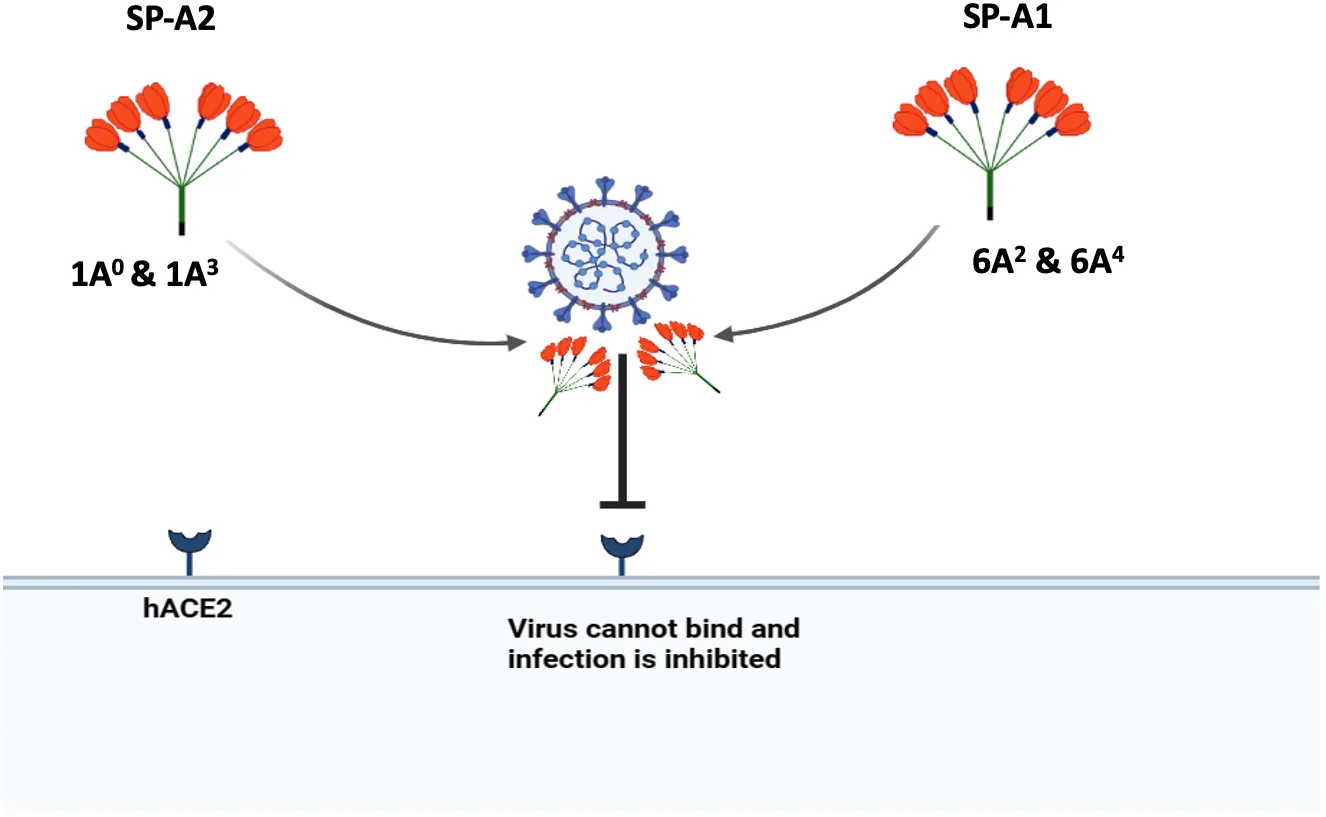
A diagram of SP-A genetic variants interaction with S protein resulting in a significant decrease in SARS-CoV-2. The SARS-CoV-2 spike protein is recognized by individual variants of human SP-A, inhibiting RBD-hACE2 interaction or viral particle aggregation to attenuate infectivity in susceptible host cells. However, the more potent interaction between the 1A^0^ variant and S protein resulted in reduced virial burden in cells compared to the 6A^2^ variant.

In this study we demonstrate that biological sex and human SP-A genetic variants as shown in **Figure 7**, differentially attenuate SARS-CoV-2-induced pulmonary and extrapulmonary disease. Based on our data that shows female 1A^3^ and 6A^4^ mice with the most attenuated ALI, we speculate that the immunomodulatory capacity of these SP-A genetic variants function interdependently with female sex difference more effectively in attenuating severe disease. Further, SP-A variants 1A^0^ and 6A^4^ and female sex most attenuate acute kidney injury, demonstrating that SP-A variants play distinct roles in different tissues. But SP-A variant alone and not sex emerged as a significant modulating factor for acute intestinal injury, suggesting that sex and SP-A differences interact and function differently in different tissues to modulate MOD. Future studies should further characterize the determine the levels of SPs in circulation and assess their interactions with host cell surface receptors in individual tissue sites, and the impact on extrapulmonary disease development post SARS-CoV-2 infection. Furthermore, it should be noted that there are several limitations in this study, including 1) a limited samples sizes used in some SP-A variants due to separate to male and female groups; 2) a few SP-A variants (proteins) used in the *in vitro* study; 3) SP-A abundance was not quantitatively compared across sex, organ, and transgenic line in the present study, and 4) a lack of mechanistic study of sex and SP-A variant interaction after SRAS-CoV-2 infection, such as how estrogen, progesterone or androgen receptors directly regulate SP-A expression or signaling.

### Implications and future directions

Collectively, our findings establish that SP-A genetic variation and biological sex are key determinants of SARS-CoV-2-induced multi-organ injury. These results have several important implications. First, they suggest that host genetic variation in innate immune proteins contributes significantly to the heterogeneity of COVID-19 outcomes. Second, they highlight the need to incorporate sex as a biological variable in studies of host–pathogen interactions. Third, they provide a rationale for exploring SP-A-based therapeutic strategies, including recombinant proteins or variant-specific interventions. Future studies should focus on elucidating the molecular mechanisms underlying SP-A variant–specific effects, including interactions with host cell receptors, downstream signaling pathways, and modulation of immune cell function. Longitudinal studies are also needed to assess the impact of SP-A variants on chronic sequelae of SARS-CoV-2 infection, including long COVID and neuroinflammatory disorders. In addition, evaluation of SP-A levels and genotypes in clinical cohorts may help validate their utility as biomarkers for disease risk and progression.

## Data Availability

The raw data supporting the conclusions of this article will be made available by the authors, without undue reservation.
